# *Nardostachys jatamansi* Extract and Nardosinone Exert Neuroprotective Effects by Suppressing Glucose Metabolic Reprogramming and Modulating T Cell Infiltration

**DOI:** 10.3390/cells14090644

**Published:** 2025-04-28

**Authors:** Congyan Duan, Weifang Lin, Mingjie Zhang, Bianxia Xue, Wangjie Sun, Yang Jin, Xiaoxu Zhang, Hong Guo, Qing Yuan, Mingyu Yu, Qi Liu, Naixuan Wang, Hong Wang, Honghua Wu, Shaoxia Wang

**Affiliations:** 1 School of Medical Technology, Tianjin University of Traditional Chinese Medicine, 10 Poyanghu Road, Jinghai Dist., Tianjin 301617, China; duancy19990514@163.com (C.D.); 17608484251@163.com (W.L.); arisaema1307@163.com (M.Z.); 18020042836@163.com (W.S.); jin1228@tjutcm.edu.cn (Y.J.); 727068105@163.com (X.Z.); yumy@tjutcm.edu.cn (M.Y.); liuqi23@tjutcm.edu.cn (Q.L.); wnaixuan021103@163.com (N.W.); wanghongsys@tjutcm.edu.cn (H.W.); 2 State Key Laboratory of Chinese Medicine Modernization, Tianjin University of Traditional Chinese Medicine, 10 Poyanghu Road, Jinghai Dist., Tianjin 301617, China; xbx1525912710@163.com (B.X.); cacti1983@163.com (H.G.); yuanxqing@tjutcm.edu.cn (Q.Y.)

**Keywords:** *Nardostachys jatamansi*, nardosinone, microglia, neuroinflammation, metabolic reprogramming, T cell infiltration, Parkinson’s disease

## Abstract

Background: *Nardostachys jatamansi* DC. (Gansong), a widely utilized herb in traditional Chinese medicine, has been historically employed in the management of various neuropsychiatric disorders. Nardosinone (Nar), a sesquiterpenoid compound, has been identified as one of the principal bioactive constituents of *N. jatamansi*. This study investigated the effects of ethyl acetate extract (NJ-1A) from *N. jatamansi* and its active constituent nardosinone on neuroinflammatory mediator release, glucose metabolic reprogramming, and T cell migration using both in vitro and in vivo experimental models. Methods: Lipopolysaccharide(LPS)-induced BV-2 microglial cells and a 1-methyl-4-phenyl-1,2,3,6-tetrahydropyridine/probenecid (MPTP/p)-induced male C57BL/6N mouse chronic model of Parkinson’s disease were applied. Results: Both NJ-1A and Nar could significantly suppress LPS-induced production of M1 pro-inflammatory factors or markers in microglia and could inhibit the glycolytic process and promote oxidative phosphorylation via the AKT/mTOR signaling pathway. Furthermore, they exhibited the capacity to attenuate chemokine release from activated microglia, consequently reducing T cell migration. In vivo experiments revealed that NJ-1A and Nar effectively inhibited microglial activation, diminished T cell infiltration, and mitigated the loss of tyrosine hydroxylase (TH)-positive dopaminergic neurons in the substantia nigra of MPTP-induced mice. Conclusions: NJ-1A and nardosinone exert neuroprotective effects through the modulation of microglial polarization states, regulation of metabolic reprogramming, and suppression of T cell infiltration.

## 1. Introduction

Microglia, serving as the resident innate immune cells of the central nervous system (CNS), play a crucial role in continuously surveilling the CNS microenvironment and mounting rapid responses to pathological changes. Based on their activation states, microglia can be polarized into two distinct phenotypes: the classically activated M1 phenotype and the alternatively activated M2 phenotype [1]. The M1 microglia is characterized by the release of pro-inflammatory cytokines and chemokines, which contribute to neuronal damage and tissue injury through both direct and indirect mechanisms. Conversely, M2 microglia exert neuroprotective effects through the secretion of anti-inflammatory factors to attenuate immune responses, facilitation of tissue repair, and regeneration via clearance of cellular debris [2]. Importantly, pharmacological modulation of the M1-to-M2 phenotypic transition represents a promising therapeutic strategy for mitigating neuronal damage and preventing the progression of neuroinflammation-related disorders.

Microglial activation induces significant alterations in cellular energy metabolism, a process referred to as metabolic reprogramming. In M1 polarized microglia, macrophages, and other immune cells under pathological conditions, glucose metabolism shifts toward aerobic glycolysis despite the presence of sufficient oxygen, accompanied by suppression of the tricarboxylic acid (TCA) cycle [3,4]. This metabolic profile closely resembles the “Warburg effect” observed in tumor cells. In contrast, the resting state and M2 polarized microglia primarily rely on oxidative phosphorylation for energy production [5,6]. Although the glycolytic pathway generates ATP less efficiently than oxidative phosphorylation, it enables rapid energy provision and facilitates the production of pro-inflammatory cytokines and reactive oxygen species (ROS) during acute inflammatory responses [7,8]. Notably, emerging evidence demonstrates that pharmacological modulation of glycolytic pathways can promote M1-to-M2 phenotypic transition and attenuate neuroinflammatory responses [4,9,10].

While the central nervous system (CNS) has traditionally been considered an immune-privileged site, emerging evidence demonstrates that pathological conditions can disrupt this privilege. The breakdown of the blood–brain barrier facilitates the infiltration of peripheral T lymphocytes into the brain parenchyma, mediated by chemotactic signals from M1 polarized microglia and other cellular sources. This process initiates an immune response following T cell recognition of antigens presented by major histocompatibility complex (MHC) molecules [11]. Notably, both CD4^+^ and CD8^+^ T cell populations have been identified in the striatum and substantia nigra of Parkinson’s disease patients and in animal models [12]. The infiltrating CD4^+^ T cells, particularly Th1 and Th17 subsets, engage with MHC class II molecules expressed on microglia [13]. Through the release of pro-inflammatory mediators such as interferon-γ [14] and interleukin-17 (IL-17) [15], these cells activate glial cells and exert detrimental effects on CNS homeostasis. Importantly, therapeutic strategies targeting CD4^+^ T cell infiltration or MHC class II expression have shown efficacy in mitigating nigrostriatal dopaminergic neuronal damage in α-synuclein overexpression models of Parkinson’s disease [16]. Brain-infiltrating CD8^+^ T cells recognize MHC class I peptide antigens and induce dopaminergic neuron apoptosis through caspase cascade activation and the release of cytotoxic mediators including granzyme and perforin [17]. Furthermore, CD8^+^ T cells can upregulate Fas ligand (FasL) expression on target neurons, thereby amplifying apoptotic signaling pathways [18,19]. Collectively, these findings highlight promising neuroprotective strategies: the modulation of glucose metabolic reprogramming to facilitate M1-to-M2 phenotypic transition, and the inhibition of T cell infiltration.

**Figure 1:**
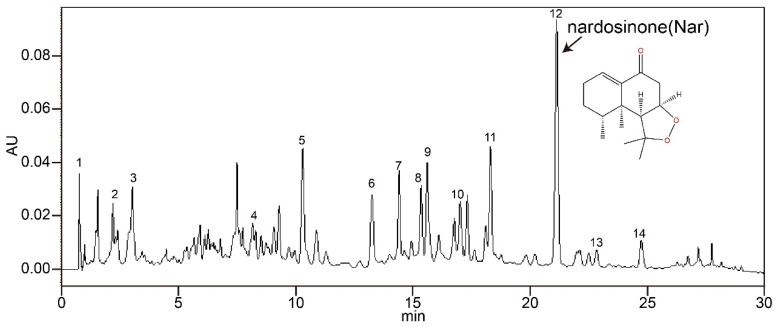
Nar is the main component of NJ-1A. Analysis by high-performance liquid chromatography (HPLC) reveals that Nar is the predominant component of NJ-1A, accounting for approximately 10.2% based on the relative peak area. The chromatographic method and the other compounds identified in NJ-1A are shown in File S1.

*Nardostachys jatamansi* DC., a widely utilized herb in Himalayan countries for centuries, has been employed in the management of various neurological and cardiovascular disorders [20]. Recent research showed that ethanol extract from a *N. jatamansi* and levodopa combination alleviates Parkinson’s disease symptoms in rats through activation of Nrf2 and inhibition of NLRP3 signaling pathways [21]. The effects of the chemical components of N. jatamansi on the molecular targets of Alzheimer’s disease have also been studied in detail [22]. NJ-1A, an ethyl acetate extract derived from *N. jatamansi*, contains nardosinone (Nar) as its principal bioactive sesquiterpenoid constituent (HPLC analysis reveals that Nar is the predominant component of NJ-1A, accounting for approximately 10.2%; see Figure 1 and File S1) [23]. Previous studies have demonstrated that nardosinone (Nar, CAS No: 23720-80-1; molecular formula: C15H22O3; molecular weight: 250.1569) exhibits neuroprotective properties, protecting SH-SY5Y cells against 6-hydroxydopamine (6-OHDA)-induced cytotoxicity and ameliorating Parkinsonian symptoms in rotenone-induced mouse models [24]. Furthermore, in rotenone-induced rat models of Parkinson’s disease, Nar has been shown to inhibit NF-κB activation, suppress neuroinflammatory mediator expression, and exert neuroprotection through modulation of slc38a2 gene expression, GABAergic synaptic transmission, and cAMP signaling pathways [25]. A recent study also showed that Nar and its metabolites might act as a potential Parkinson adjutant to enhance the efficacy of levodopa via the intestinal flora [26]. Despite these advances, the effects of this herbal compound on the metabolic reprogramming of activated microglia and their interactions with brain-infiltrating T lymphocytes remain poorly characterized.

This study aimed to investigate the therapeutic potential and mechanisms of NJ-1A and Nar in Parkinson’s disease by using two well-established models: an in vitro lipopolysaccharide (LPS)-induced microglial model, and an in vivo 1-methyl-4-phenyl-1,2,3,6-tetrahydropyridine/probenecid (MPTP/p)-induced mouse chronic model of Parkinson’s disease. MPTP can cause damage to dopaminergic neurons in mice. This damage mimics the pathological changes observed in Parkinson’s disease patients, rendering it an outstanding model for researching the disease’s pathogenesis [27]. Our findings demonstrate that both NJ-1A and Nar confer neuroprotection through modulation of microglial glucose metabolic reprogramming and inhibition of T cell infiltration. Importantly, this study provides novel insights into the neuroprotective mechanisms of herbal medicine by elucidating its impact on adaptive immune responses in Parkinson’s disease pathogenesis.

## 2. Materials and Methods

### 2.1. Animals

Sixty male SPF C57BL/6N mice (8 weeks old) were acquired from Beijing Charles River Biotechnology Co., Ltd. (Beijing, China). Mice were raised in an environment with a temperature of 24 ± 2 °C and a relative humidity of 35 ± 5%. They had free access to food and water and were adaptively reared for one week prior to MPTP treatment. All experimental procedures were conducted in accordance with the guidelines approved by the Institutional Animal Care and Use Committee of Tianjin University of Traditional Chinese Medicine (Approval Number: TCM-LAEC2023201d1536).

### 2.2. NJ-1A and Nar

*N. jatamansi* plants used in this study were kindly provided and authenticated by Prof. Honghua Wu (State Key Laboratory of Chinese Medicine Modernization, Tianjin University of Traditional Chinese Medicine, Tianjin, China). The extraction procedures of NJ-1A were as follows: take 30 g of *N. jatamansi* powder passed through a 50-mesh sieve, add 600 milliliters of analytical grade methanol, and extract ultrasonically two times 2 h apart. Then, filter the resultant solution using 15–20 μm medium-speed filter paper, and concentrate to dryness under reduced pressure. Subsequently, suspend the extract in 10-times distilled water (*w*/*v*) and extract three times with an equal volume of ethyl acetate. After similar filtration and concentration procedures, the liposoluble component NJ-1A of *N. jatamansi* can be obtained. HPLC analysis reveals that Nar is the predominant component of NJ-1A, accounting for approximately 10.2% based on the relative peak area (Figure 1). The chromatographic method and the compounds identified in NJ-1A (including their name, content, retention time, etc.) are shown in File S1.

Nar (CAS No. 23720-80-1, (purity ≥ 98%)) was purchased from Chengdu Purifa Technology Co., Ltd. (Chengdu, China). NJ-1A and Nar were dissolved in dimethyl sulfoxide (DMSO, for in vitro experiments) or corn oil (for in vivo experiments).

### 2.3. Cell Culture and Treatment

The mouse BV-2 microglial cell line was obtained from Cell Resource Center, Institute of Basic Medical Sciences, Chinese Academy of Medical Sciences, and cultured in Dulbecco’s modified Eagle’s medium (DMEM, Gibco, Waltham, MA, USA) supplemented with 10% fetal bovine serum (Gibco), 100 U/mL penicillin, and 100 µg/mL streptomycin (Viva Cell, Shanghai, China). It was divided into the control group, the LPS group, the positive control minocycline group (Mino, Sigma-Aldrich, St Louis, MO, USA), the groups of nardosinone (Nar) at 2.5, 5, and 10 μM, and the groups of NJ-1A at 5, 10, and 25 μg/mL. For the NJ-1A and Nar groups, the microglia were pretreated with NJ-1A or Nar for 30 min, and then the cells were treated with LPS and NJ-1A or Nar for different periods. The LPS group was only treated with LPS and solvent (DMSO, Sigma-Aldrich, St Louis, MO, USA). The exact number of experimental units, determined based on experience, is specified in the figure legends. For each experimental group, no data points were excluded.

### 2.4. Cell Viability Assay and Detection of Nitric Oxide and Cytokines

BV-2 cells were seeded in 48-well plates at a density of 1.6 × 10^5^ cells per well. The microglia were pretreated with NJ-1A or Nar for 30 min, and then the cells were treated with LPS and the drugs for 24 h. The supernatant of BV-2 cells was aspirated to measure the contents of NO, cytokines, and chemokines by commercial kits. (NO, Beyotime Biotechnology, Haimen, China, S0021S; TNF-α, IL-6 Elisa kits and Mouse Chemokine Array kit, R&D Systems Inc., Minneapolis, MN, USA, MTA00B, M6000B and ARY020; CCL5, CXCL 10 Elisa kits, elabscience, Wuhan, China, E-EL-M0009c and E-EL-M0021). Cell viability was quantified by CCK-8 assay(Beyotime Biotechnology, Haimen, China, C0037).

### 2.5. Real-Time Reverse Transcription-Polymerase Chain Reaction (Real-Time RT-PCR)

BV-2 cells were seeded in 6-well plates at a density of 8 × 10^5^ cells per well. After treatment with LPS and/or drugs for 8 h (for inflammatory factor indicators) and 24 h (for glycolytic indicators and chemokine indicators), the expression of related mRNAs was detected by quantitative RT-PCR using a kit (Applied Biosystems, Waltham, MA, USA, A25742). The primer sequences are shown in File S1. RT-negative controls were also used to monitor DNA contamination.

### 2.6. Reactive Oxygen Species (ROS)

BV-2 cells were seeded in 12-well plates at a density of 3 × 10^5^ cells per well. After treatment with LPS and/or drugs for 24 h, the cells were washed and collected. The cells were incubated with a DCFH probe (10 μM) in the dark for 1 h. After being washed with PBS, the levels of ROS were analyzed by a flow cytometer and a fluorescence microscope.

### 2.7. EACR and OCR

A XF-96 extracellular flux analyzer (Agilent, Santa Clara, CA, USA) was used to detect the extracellular acidification rate (ECAR) and oxygen consumption rate (OCR) in LPS-induced BV-2 cells (1 × 10^4^ cells per well). According to the kit instructions, the response of ECAR to the stimulation of 10 mM glucose, 1 µM oligomycin, and 50 mM 2-deoxy-D-glucose (all from Agilent, Santa Clara, CA, USA, 103020-100) was evaluated. Then, OCR analysis was carried out for 1.5 µM oligomycin, 1 µM FCCP, and 0.5 µM rotenone/antimycin A (all from Agilent, Santa Clara, CA, USA, 103020-100).

### 2.8. Western Blot

BV-2 cells were seeded in 6-well plates at a density of 8 × 10^5^ cells per well. After the cells were treated with LPS and/or drugs for 1 h or 24 h, the total protein was extracted. Western blot analysis was performed using the primary antibody and horseradish peroxidase (HRP)-coupled secondary antibody. Primary antibodies were purchased from Cell Signaling Technology (Danvers, MA, USA; β-actin (3700S), p65 (8242S), p-p65 (3033S), iκB-α (4812S), p-iκB-α (2859S), mTOR (29182S), AMPK (5831S), p-AMPK (2535S), TH (58844S), Iba-1 (17198S)) and Thermo Fisher Scientific (Waltham, MA, USA; iNOS (14-5920-82)).

### 2.9. The Effect of Conditioned Medium on Migration of CTLL-2 Cells

BV-2 microglial cells were plated in 48-well culture plates at a density of 1.6 × 10^5^ cells per well. Following a 30 min pretreatment with drugs, cells were stimulated with LPS and incubated for 24 h. Culture supernatants were subsequently collected and transferred to the lower chambers of Transwell plates. CTLL-2 cells, after serum starvation for 30 min, were seeded in the upper chambers at a density of 8 × 10^4^ cells per well. The migration assay was conducted for 24 h at 37 °C in a humidified 5% CO_2_ atmosphere. Migrated CTLL-2 cells in the lower chambers were quantified using flow cytometry.

### 2.10. MPTP/p-Induced Chronic Mouse Parkinson Model and Treatment

Sixty male healthy SPF C57BL/6N mice (8 weeks old, weight 22 to 24 g) were randomly allocated into six groups by using a random number table, each consisting of ten mice: (1) Control group, (2) MPTP group, (3) Nar-5 mg/kg group, (4) Nar-50 mg/kg group, (5) NJ-1A-10 mg/kg group, and (6) NJ-1A-100 mg/kg group. A MPTP/p-induced chronic mouse Parkinson model was applied [27]. On the day of model establishment, the mice were first intraperitoneally injected with probenecid sodium solution at a dose of 250 mg/kg, and 30 min later, MPTP solution at a dose of 30 mg/kg was administered intraperitoneally. The mice in the Control group were injected intraperitoneally with an equivalent volume of saline. Two hours after the intraperitoneal injection of MPTP and on the subsequent day, NJ-1A at doses of 10 mg/kg and 100 mg/kg, as well as Nar at doses of 16 mg/kg and 80 mg/kg, were administered via intraperitoneal injection. Concurrently, the mice in the Control and MPTP groups were intraperitoneally injected with the corresponding volume of corn oil. The administration of MPTP and/or drugs to the mice was carried out every 3.5 days (i.e., twice a week) for a period of 5 weeks. In total, MPTP was administered ten times cumulatively, and the drugs were administered twenty times. The order of administration for each drug administration treatment in each group was determined using a randomization method. Before performing an intraperitoneal injection on a mouse, the abdominal area was disinfected with an alcohol swab, and a relatively small 27 G needle was used to inject the substance slowly. The animal was soothed both before and after the injection. The mice were anesthetized with 2% sodium pentobarbital solution before euthanasia. The experiment was to be terminated if more than 20% of the mice injected with MPTP lost more than 20% of their initial body weight within a week or showed severe motor function impairment (such as hunched back, dragging limbs, unsteady gait, and muscle rigidity). The body weight and motor status of the mice was monitored twice a week. During the experiment, no adverse events occurred. In fact, at the end of the experiment, the body weight of all the mice increased by 5 to 6 g (see Section 3.8). All the animals were included during the experiment. For each experimental group, no animals or data points were excluded.

### 2.11. Immunohistochemistry and Immunofluorescence

The mice were anesthetized and perfused. The entire brains were successively placed in paraformaldehyde solution and sucrose solution for external fixation and dehydration. Brain tissue was cryosectioned, and coronal sections were taken from the striatum and substantia nigra, with a thickness of 40 μm. Then, routine immunofluorescence and immunofluorescence experiments were carried out with specific primary antibodies (TH and Iba-1, Cell Signaling Technology (Danvers, MA, USA, 58844S and 17198S; CD4 and CD8, Thermo Fisher Scientific (Waltham, MA, USA, 14-0041-82 and 14-0081-82) and secondary antibodies (ZSGB-BIO, Beijing, China, ZLI-9018).

### 2.12. Rotarod

The mice were subjected to rotarod training on the 34th and 35th days. On the 36th day, the rotarod test was carried out. The speed of the rotarod gradually increased from 4 r/min to 30 r/min in 300 s. The latency period was recorded as the duration for each mouse to fall off the rod.

### 2.13. Statistical Analysis

All researchers were aware of the group allocation, except for the individual (B.X.) responsible for analyzing and quantifying the results of Western Blot and immunohistochemistry/immunofluorescence tests. All experimental data were tested for homogeneity of variance and normality. Data were presented as mean ± standard deviation and analyzed with SPSS 26.0. For multiple-group data comparison, use a one-way ANOVA LSD test for homogeneous variance and non-parametric tests for heterogeneous variance. A *p*-value < 0.05 indicates statistical significance. We carried out a power analysis based on the results of the expression level of tyrosine hydroxylase, the crucial pharmacodynamic measure. The calculated power value was 0.8319.

## 3. Results

### 3.1. NJ-1A and Nar Can Inhibit the Production of Pro-Inflammatory Factors in BV-2 Cells Induced by LPS

**Figure 2:**
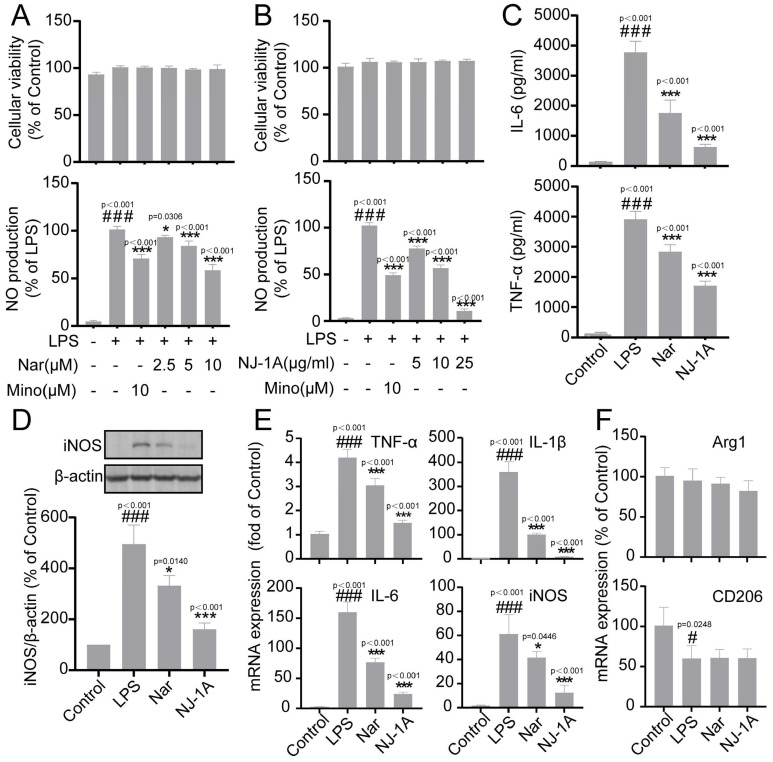
NJ-1A and Nar can inhibit the production of pro-inflammatory factors in microglia induced by LPS. (**A**–**C**) NJ-1A and Nar can inhibit the production of NO, IL-6, and TNF-α in LPS-induced BV-2 cells without affecting cell viability, *n* = 5; (**D**) NJ-1A and Nar can inhibit the expression of iNOS protein, *n* = 3; (**E**,**F**) NJ-1A and Nar can inhibit the mRNA expression of pro-inflammatory factors but do not inhibit the mRNA expression of anti-inflammatory cytokines, *n* = 4. Data represent the mean ± SD; vs. Control, # *p* < 0.05, ### *p* < 0.001; vs. LPS, * *p* < 0.05, *** *p* < 0.001. NJ-1A: Ethyl acetate extract of *N. jatamansi*, Nar: nardosinone, LPS: Lipopolysaccharide, MINO: Minocycline.

First, we investigated the inhibitory effects of NJ-1A and Nar on LPS-induced inflammatory factors in microglia. The results demonstrated that minocycline, used as a positive control, significantly inhibited the production of pro-inflammatory factors (nitric oxide (NO), interleukin-6 (IL-6), and tumor necrosis factor-α (TNF-α)) induced by LPS. Both Nar and NJ-1A potently inhibited the production of the aforementioned cytokines without affecting cell viability (Figure 2A–C). We also detected the expression of inducible nitric oxide synthase (iNOS), which is the key enzyme catalyzing NO production. The results showed that, after treatment with NJ-1A and Nar separately, the expression of iNOS decreased significantly compared to the LPS group (Figure 2D). Treatments with NJ-1A and Nar could also markedly reduce the mRNA expressions of these pro-inflammatory factors (Figure 2D). However, treatments with NJ-1A and Nar had no impact on the mRNA expressions of M2 anti-inflammatory factors Arginase 1 (Arg1) and CD206 (Figure 2F).

### 3.2. NJ-1A and Nar Inhibit the Release of Reactive Oxygen Species (ROS) in Microglia Induced by LPS

**Figure 3:**
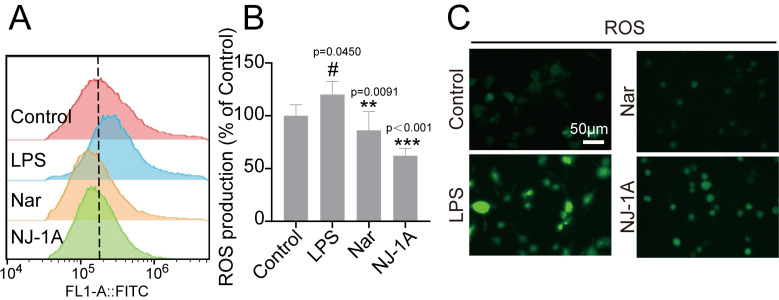
NJ-1A and Nar can inhibit the generation of ROS in BV-2 cells induced by LPS. (**A**,**B**) After BV-2 cells were stimulated with LPS for 24 h, the content of ROS inside the cells was detected by flow cytometry, *n* = 4. (**C**) Fluorescence microscopic images of BV-2 cells treated with LPS and/or NJ-1A and Nar after incubation with DCFH probe for 24 h. Data represent the mean ± SD; vs. Control, # *p* < 0.05; vs. LPS, ** *p* < 0.01, *** *p* < 0.001.

Inflammatory factors released by activated microglia promote the production of reactive oxygen species (ROS) by interfering with the normal operation of the electron transport chain [28]. A substantial body of literature has demonstrated that oxidative stress plays a crucial role in the degeneration of dopaminergic neurons and exacerbates the pathological progression of Parkinson’s disease [29]. We employed DCFH-DA fluorescent probes to examine the production of ROS in microglia. The results of flow cytometry indicated that 24 h after LPS stimulation, the intracellular ROS content in BV-2 cells increased significantly compared to the Control group. However, treatment with NJ-1A or Nar could markedly reduce the ROS concentration (Figure 3A,B). The fluorescence microscopy data further corroborated these findings (Figure 3C).

### 3.3. NJ-1A and Nar Inhibit the Phosphorylation Levels of IκB-α and NF-κB p65

**Figure 4:**
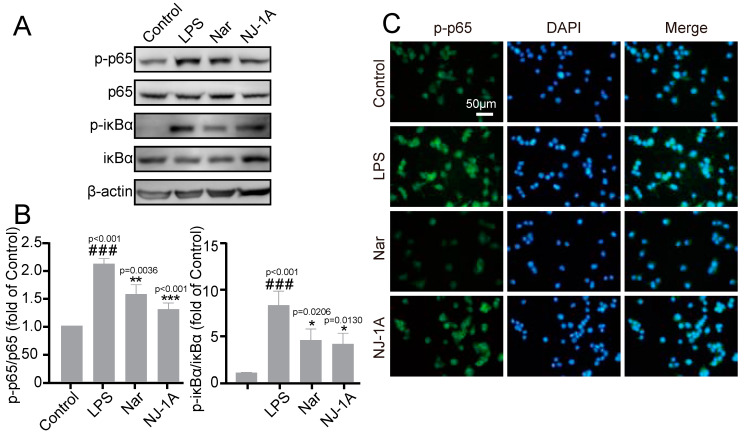
NJ-1A and Nar can inhibit the phosphorylation levels of IκB-α and NF-κB p65 in BV-2 cells after LPS stimulation. (**A**,**B**) The results of Western blot showed that NJ-1A and Nar could inhibit the phosphorylation of IκB-α and NF-κB p65, *n* = 4. (**C**) The results of fluorescence microscopy of the cells showed that NJ-1A and Nar could inhibit the phosphorylation of NF-κB p65. Data represent the mean ± SD; vs. Control, ### *p* < 0.001; vs. LPS, * *p* < 0.05, ** *p* < 0.01, *** *p* < 0.001.

Subsequently, we investigated whether NJ-1A and Nar could inhibit the activation of NF-κB, a key transcription factor regulating the expression of pro-inflammatory mediators in LPS-stimulated microglia. The results (Figure 4A,B) revealed that, upon 1 h stimulation of BV-2 microglial cells with LPS, the phosphorylation levels of both IκB-α and NF-κB p65 within the cells increased significantly. However, treatment with NJ-1A and Nar led to a significant reduction in the phosphorylation of these proteins. The immunofluorescence results further validated the above-mentioned findings (Figure 4C).

### 3.4. NJ-1A and Nar Can Inhibit the Glycolytic Process of LPS-Induced BV-2 Cells and Promote Oxidative Phosphorylation

**Figure 5:**
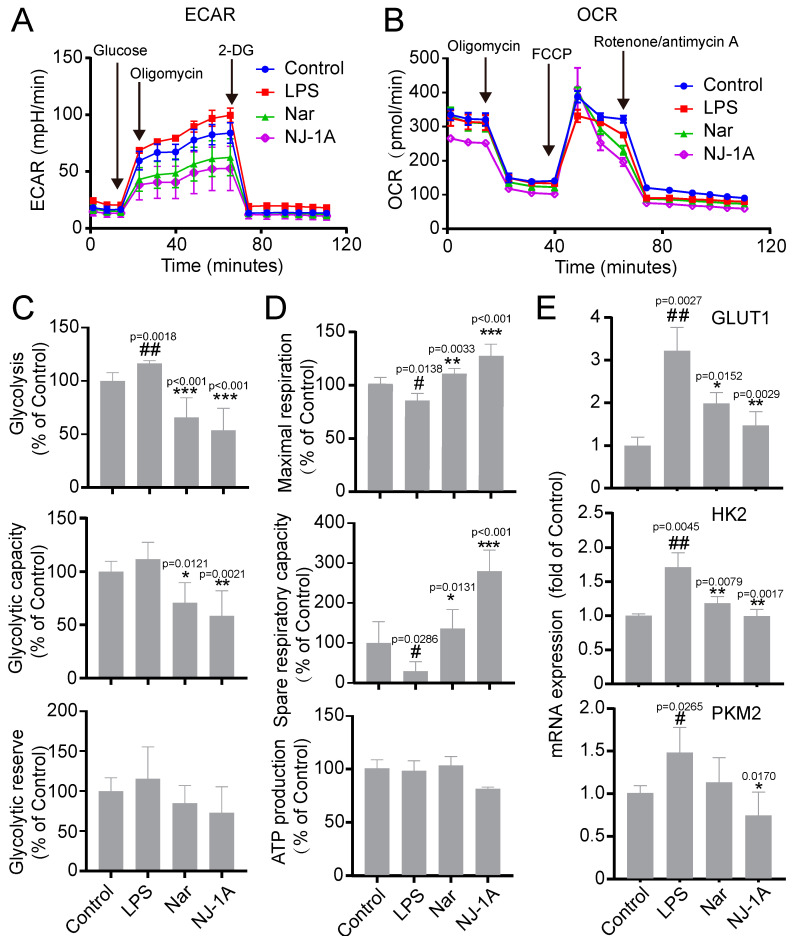
NJ-1A and Nar can reverse the glucose metabolic reprogramming of LPS-induced BV-2 cells. (**A**,**C**) NJ-1A and Nar can inhibit the increase of ECAR in BV-2 cells, *n* = 5, (**B**,**D**) and can reverse the decrease of OCR in BV-2 cells, *n* = 4. (**E**) NJ-1A and Nar inhibited the mRNA expression of key glycolytic enzymes, *n* = 4. Data represent the mean ± SD; vs. Control, # *p* < 0.05, ## *p* < 0.01; vs. LPS, * *p* < 0.05, ** *p* < 0.01, *** *p* < 0.001.

To investigate the effects of NJ-1A and Nar on the glucose metabolic reprogramming of M1-phenotype microglia, we measured the extracellular acidification rate (ECAR, a direct indicator of glycolysis) and oxygen consumption rate (OCR, a direct indicator of mitochondrial respiration) of the cells using the Seahorse XF96 energy metabolism system. We observed that 24 h after LPS treatment, the ECAR level of microglia increased, and the glycolytic capacity under basal conditions also rose (Figure 5A,C). However, treatment with NJ-1A and Nar decreased the ECAR level, glycolytic capacity, and glycolytic reserve under basal conditions (Figure 5A,C). OCR detection data showed that 24 h after LPS stimulation, the oxygen consumption rate of microglia was significantly reduced, and both the maximal respiration of the cells and the difference between maximal and basal respiration (spare respiratory capacity) were significantly lower (Figure 5B,D). Nevertheless, the administration of NJ-1A and Nar alleviated this inhibitory mechanism (Figure 5B,D). This indicates that NJ-1A and Nar can inhibit the glycolytic capacity of M1 pro- inflammatory phenotype microglia and promote their oxidative phosphorylation capacity.

Subsequently, we evaluated the mRNA expression of key glycolytic enzymes. After LPS stimulation, the mRNA expression of glycolytic enzymes, including glucose transporter 1 (GLUT1), hexokinase 2 (HK2), and pyruvate kinase M2 (PKM2), was upregulated. In contrast, NJ-1A and Nar decreased the expression of these enzymes (Figure 5E).

### 3.5. NJ-1A and Nar Can Suppress the Production of Chemokines in LPS-Induced BV-2 Cells and Further Inhibit the Migration of CTLL-2 Cells

**Figure 6:**
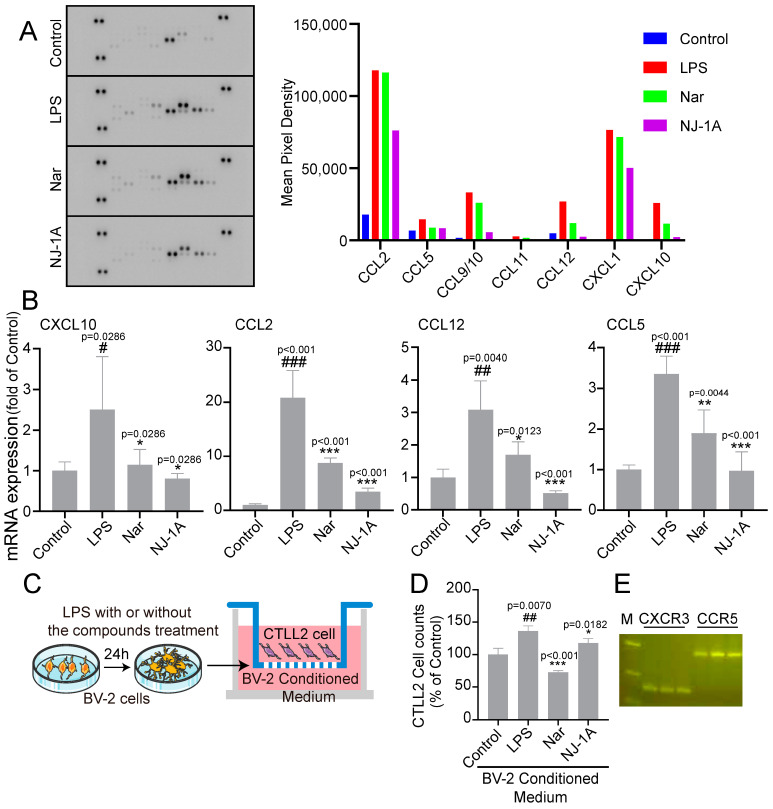
NJ-1A and Nar can inhibit the production of chemokines and the migration of T lymphocyte line CTLL-2. (**A**) The results of the chemokine protein chip showed that NJ-1A and Nar could inhibit the secretion of several chemokines. (**B**) NJ-1A and Nar inhibited the mRNA expression of CXCL10, CCL2, CCL12, and CCL5 chemokines, *n* = 4. (**C**) Schematic diagram of the Transwell experiment. (**D**) The results of flow cytometry showed that NJ-1A and Nar could inhibit the migration of CTLL-2 cells, *n* = 3. (**E**) RT-PCR results showed that CTLL-2 cells expressed CXCR3 and CCR5. Data represent the mean ± SD; vs. Control, # *p* < 0.05, ## *p* < 0.01, ### *p* < 0.001; vs. LPS, * *p* < 0.05, ** *p* < 0.01, *** *p* < 0.001.

To clarify the types of chemokines secreted by activated microglia and determine whether NJ-1A and Nar can inhibit their production, we first employed a protein microarray to assess the expression levels of 25 different chemokines. The results indicated that the expression of seven LPS-induced chemokines, namely C-C motif chemokine ligand 2 (CCL2), CCL5, CCL9/10, CCL11, CCL12, C-X-C motif chemokine ligand 1 (CXCL 1), and CXCL10, was downregulated following treatment with NJ-1A and Nar (Figure 6A). Subsequently, we further examined the mRNA expression of CXCL10, CCL2, CCL12, and CCL5 in BV-2 cells. The results were consistent with those obtained from the protein microarray (Figure 6B).

To verify whether the inhibitory effects of NJ-1A and Nar on the aforementioned chemokines could translate into an inhibitory effect on lymphocyte migration, we utilized the Transwell assay to evaluate the impact of the conditioned medium from BV-2 cells treated with LPS and/or NJ-1A/Nar on the migration of the mouse T lymphocyte cell line CTLL-2 (Figure 6C). The conditioned medium from LPS-stimulated BV-2 cells significantly increased the number of CTLL-2 cells migrating through the Transwell chamber. In contrast, the conditioned medium from BV-2 cells treated with NJ-1A and Nar led to a marked reduction in the number of CTLL-2 cells passing through the chamber (Figure 6D).

The binding of chemokines to their corresponding receptors on the surface of T cells is essential for T cell migration [30]. We previously confirmed that four chemokines, CCL2, CCL5, CCL12, and CXCL10, are highly expressed by M1 polarized pro-inflammatory microglia. These chemokines can bind to chemokine receptors, including CXC chemokine receptor 3 (CXCR3) and C-C motif chemokine receptor 5 (CCR5). Next, we identified the types of receptors expressed by mouse CTLL-2 cells. The findings (Figure 6E) demonstrated that mouse CTLL-2 lymphocytes indeed express the chemokine receptors CXCR3 and CCR5, suggesting that CTLL-2 cells are capable of recognizing and binding chemokines produced by microglia.

### 3.6. NJ-1A and Nar Can Inhibit the Expression of MHCII in LPS-Induced BV-2 Cells

**Figure 7:**
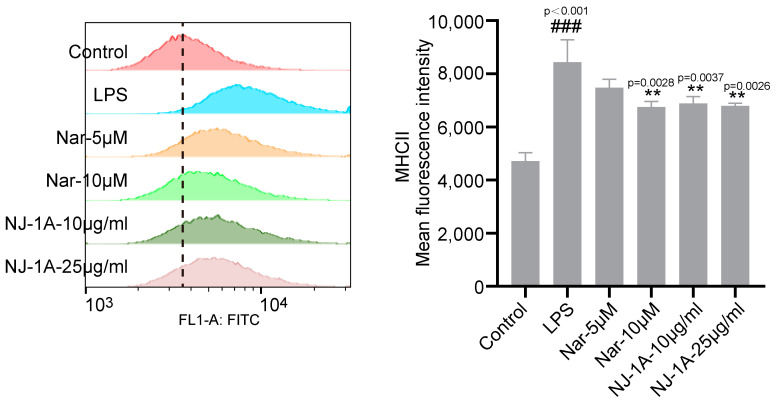
NJ-1A and Nar could inhibit the expression of MHCII in BV-2 cells induced by LPS, *n* = 4. Data represent the mean ± SD; vs. Control, ### *p* < 0.001; vs. LPS, ** *p* < 0.01.

Major histocompatibility complex class II (MHCII) is expressed on the surface of activated microglia and plays a crucial role in antigen presentation to T cells. This process mediates the infiltration of CD4^+^ T cells, thereby exacerbating neuroinflammation and neuronal damage. We employed flow cytometry to investigate the effects of NJ-1A and Nar on MHCII expression in activated microglia. The results showed that a 24 h induction with LPS significantly upregulated MHCII expression in BV-2 cells, whereas treatment with NJ-1A and Nar markedly downregulated MHCII expression (Figure 7).

### 3.7. NJ-1A and Nar Can Inhibit the Secretion of Chemokines and Inflammatory Factors in Activated BV-2 Cells Through the AKT/mTOR Signaling Pathway

**Figure 8:**
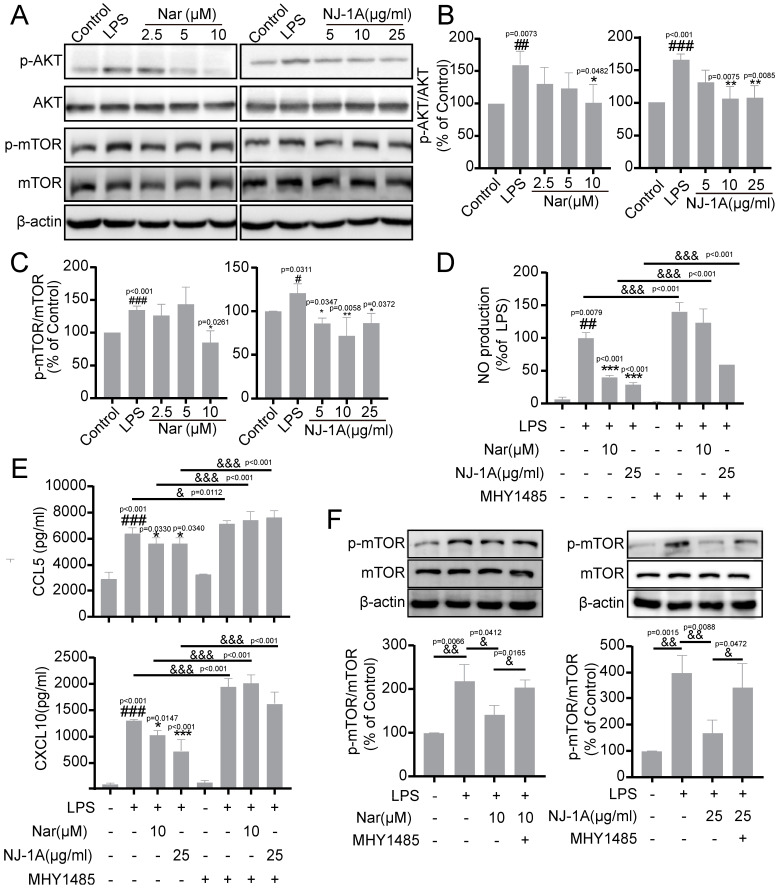
NJ-1A and Nar inhibit the secretion of chemokines and inflammatory factors in activated BV-2 cells through the AKT/mTOR signaling pathway. (**A**–**C**) NJ-1A and Nar inhibited the phosphorylation of AKT and mTOR, *n* = 3. (**D**,**E**) The mTOR agonist MHY1485 abolished the inhibitory effects of NJ-1A and Nar on NO, CCL5, and CXCL10, *n* = 5. (**F**) The mTOR agonist MHY1485 eliminated the effect of NJ-1A and Nar on the phosphorylation of mTOR, *n* = 3. Data represent the mean ± SD; vs. Control, # *p* < 0.05, ## *p* < 0.01, ### *p* < 0.001; vs. LPS, * *p* < 0.05, ** *p* < 0.01, *** *p* < 0.001, & *p* < 0.05, && *p* < 0.01, &&& *p* < 0.001.

Protein kinase B (AKT) activates the mammalian target of rapamycin (mTOR) via phosphorylation and is involved in metabolism, T cell-mediated immune responses, and the aging process [31]. Subsequently, we explored the effects of NJ-1A and Nar on the AKT/mTOR signaling pathway. The results (Figure 8A–C) demonstrated that both NJ-1A and Nar suppressed LPS-induced phosphorylation of AKT and mTOR. Moreover, MHY1485, a cell-permeable mTOR agonist, was able to reverse the inhibitory effects of NJ-1A and Nar on the secretion of nitric oxide (NO), CCL5, and CXCL10 (Figure 8D,E). Additionally, we found that MHY1485 could alleviate the impact of NJ-1A and Nar on mTOR phosphorylation (Figure 8F). Collectively, these findings suggest that NJ-1A and Nar can inhibit the secretion of chemokines and inflammatory factors in activated BV-2 cells through the AKT/mTOR signaling pathway.

### 3.8. NJ-1A and Nar Can Ameliorate the Damage of Dopaminergic Neurons

**Figure 9:**
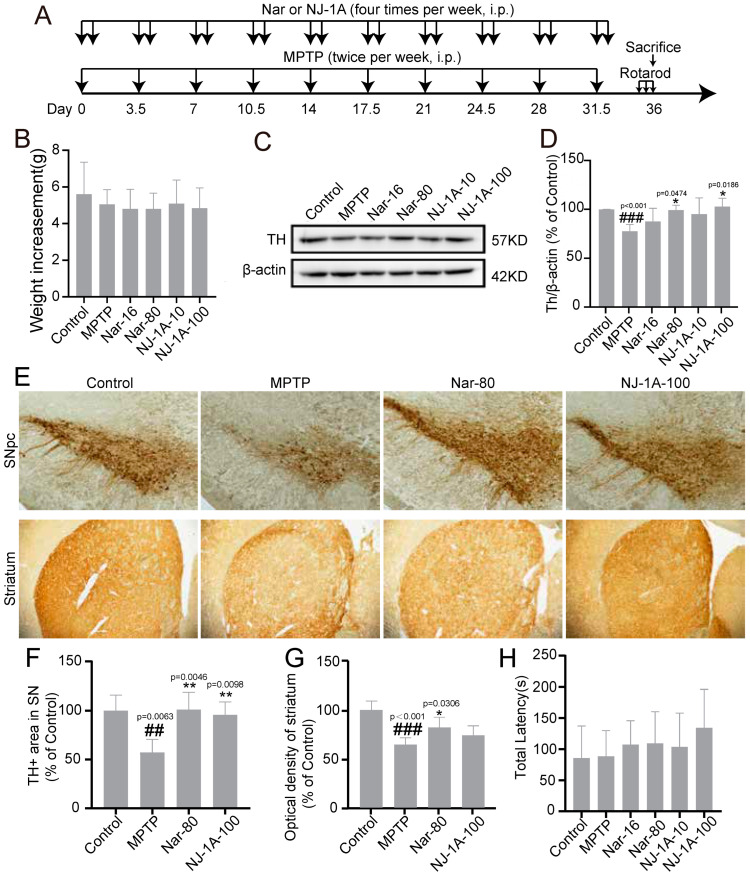
NJ-1A and Nar can ameliorate the damage of dopaminergic neurons in the MPTP-induced mouse model of Parkinson’s disease. (**A**) In vivo experimental flow chart. (**B**) The weight gain of mice at the end of the experiment, *n* = 10. (**C**,**D**) Western blot results showed that NJ-1A and Nar can reverse the reduction of TH expression in the ventral midbrain of the mouse model of Parkinson’s disease, *n* = 4. (**E**–**G**) Immunohistochemistry results showed that NJ-1A and Nar can reverse the reduction of TH-positive cells in the substantia nigra and striatum of the mouse model of Parkinson’s disease, *n* = 4. (40x magnification) (**H**) The rotarod test. Data represent the mean ± SD; vs. Control, ## *p* < 0.01, ### *p* < 0.001; vs. MPTP, * *p* < 0.05, ** *p* < 0.01.

We utilized MPTP-induced chronic Parkinson’s disease mouse models to explore the neuroprotective effects of NJ-1A and Nar (Figure 9A). During the entire experiment, the body weight of the mice in each group increased by 5–6 g, and there was no significant difference in the increased values among the groups (Figure 9B). The results indicated that the expression level of tyrosine hydroxylase (TH, a major marker of dopaminergic neurons in the ventral midbrain) in the brains of MPTP-induced mice was significantly decreased. After treatment with different doses of NJ-1A and Nar, the expression of TH in the mouse brains increased (Figure 9C,D). Moreover, immunohistochemical staining results also revealed a substantial reduction in the number of TH-positive cells in the substantia nigra and striatum of the brains of chronic Parkinson’s disease mice. Following the administration of the drugs NJ-1A and Nar, the number of TH-positive cells increased (Figure 9E–G). However, the MPTP group did not show impairments in rotarod tests compared to the control group, and the drug-administered group did not exhibit any improvement effects (Figure 9H).

### 3.9. NJ-1A and Nar Can Inhibit the Activation of Microglia in the Brains of Mouse Models of Parkinson’s Disease

**Figure 10:**
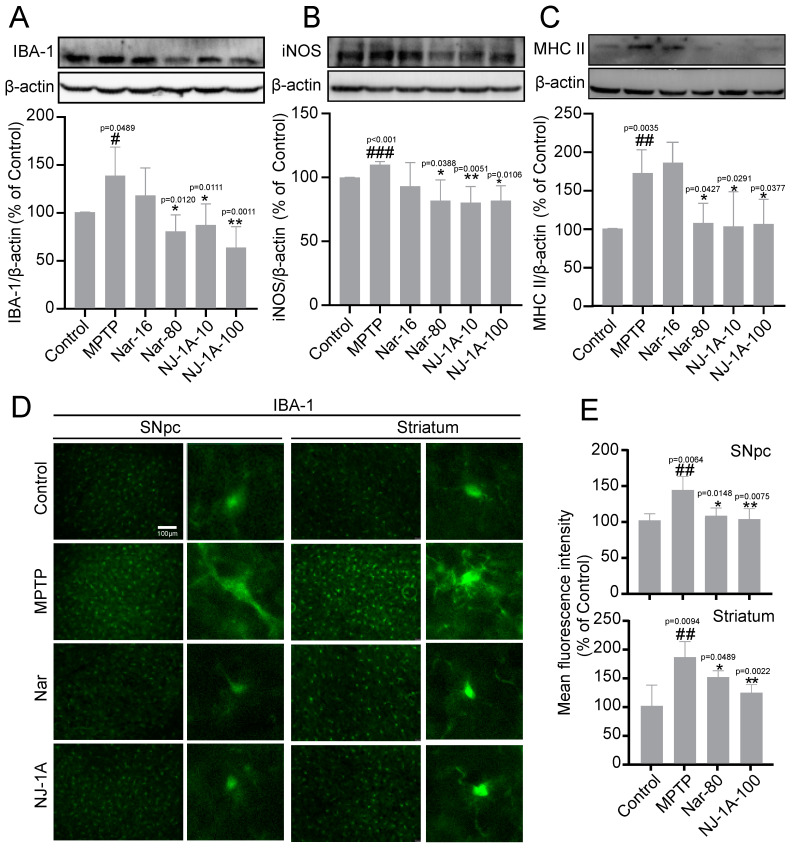
NJ-1A and Nar can inhibit the activation of microglia in the brains of mice in models of Parkinson’s disease induced by MPTP. (**A**–**C**) Western blot results showed that NJ-1A and Nar can inhibit the expression of activated microglial markers IBA-1, iNOS, and MHCII in the ventral midbrain, *n* = 4; (**D**,**E**) Immunofluorescence results demonstrated that NJ-1A and Nar can suppress the expression of the activated microglial marker Iba1 in the substantia nigra and striatum, *n* = 4. Data represent the mean ± SD; vs. Control, # *p <* 0.05, ## *p <* 0.01, ### *p <* 0.001; vs. MPTP, * *p* < 0.05, ** *p <* 0.01. Scale bar, 100 μm.

Subsequently, we investigated the effects of NJ-1A and Nar on microglial activation in the brains of MPTP-induced chronic Parkinson’s disease mouse models. In the ventral midbrain of mice in the MPTP model group, the expression levels of IBA-1 (ionized calcium-binding adaptor molecule 1, an activation marker of microglia), inducible nitric oxide synthase (iNOS), and major histocompatibility complex class II (MHCII) were significantly up-regulated (Figure 10A–C). In contrast, in the drug-treated mice, the protein expression of IBA-1 was maintained at the control levels, and the expression levels of iNOS and MHCII were reduced. Immunofluorescence experiments also confirmed the activation of microglia in the substantia nigra and striatum, as well as the effects of NJ-1A and Nar on IBA-1 expression (Figure 10D,E).

### 3.10. NJ-1A and Nar Can Inhibit the Infiltration of T Cells in the Brains of Mice in Models of Parkinson’s Disease

**Figure 11:**
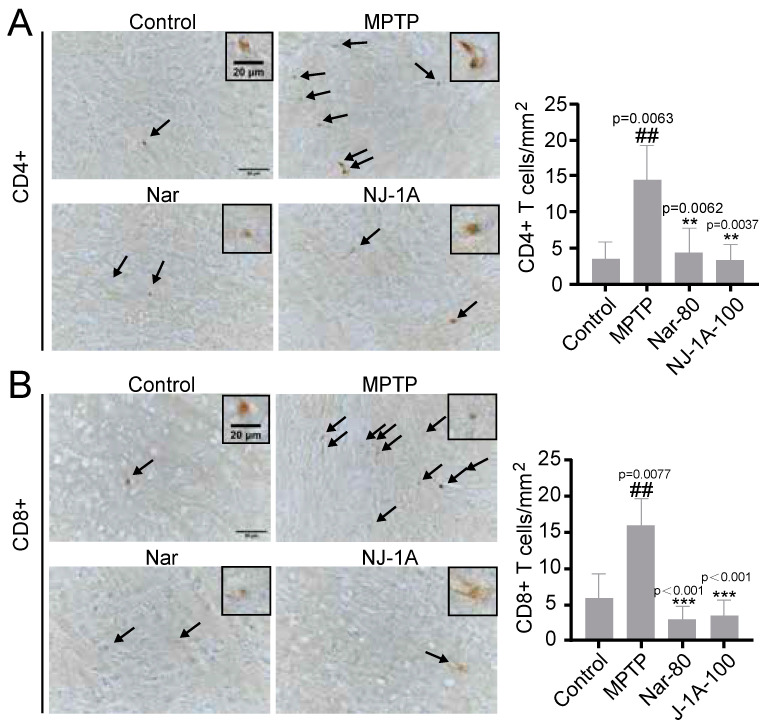
NJ-1A and Nar can reduce the number of CD4^+^ T (**A**) and CD8^+^ T **(B**) cells in the substantia nigra, *n* = 4. The black arrows indicate the positive cells, and the frames indicate the magnified positive cells. Data represent the mean ± SD; vs. Control, ## *p* < 0.01; vs. MPTP, ** *p* < 0.01, *** *p <* 0.001.

Subsequently, we investigated the effects of NJ-1A and Nar on the infiltration of CD4^+^ T cells and CD8^+^ T cells in the brains of MPTP-induced chronic Parkinson’s disease model mice. The numbers of both CD4^+^ T cells and CD8^+^ T cells in the substantia nigra of the brains of MPTP-induced mice were significantly increased. Conversely, the infiltration numbers of CD4^+^ T cells and CD8^+^ T cells in the NJ-1A and Nar treated groups were significantly decreased (Figure 11A,B).

## 4. Discussion

Parkinson’s disease (PD) ranks as the second most prevalent neurodegenerative disorder globally, afflicting a substantial number of patients with an upward trend in incidence [32]. PD patients commonly present with motor behavioral impairments along with non-motor symptoms. These manifestations significantly deteriorate their quality of life, imposing a psychological burden on both the patients and their families. First-line medications, such as levodopa, are incapable of arresting the disease progression or preventing a variety of side effects [33]. Given its complex etiology, influenced by environmental, genetic, and other factors, and the fact that the majority of patients are sporadic, the exact cause of PD remains incompletely understood at present [29]. The existing research indicates that over-activated microglia contribute to the development of Parkinson’s disease. Autopsy findings reveal the accumulation of a large number of activated M1-phenotype microglia in the lesioned brain tissues of PD patients and the detection of numerous inflammatory factors [34].

Microglia play a crucial role in the brain’s innate immune response during the development and progression of Parkinson’s disease. Based on their activation states, microglia can be phenotypically categorized into the M1 (pro-inflammatory) and M2 (anti-inflammatory and reparative) phenotypes [35,36]. It has been firmly established that modulating the polarization of microglia from the M1 to the M2 phenotype can confer neuroprotective effects [2]. Our findings demonstrate that both NJ-1A and Nar decreased the expression of pro-inflammatory factors, including NO, TNF-α, and IL-6, while having no impact on the expression of anti-inflammatory factors like CD206 and Arg1 (Figure 1, Figure 2 and Figure 3). These results suggest that NJ-1A and Nar can facilitate the transition of microglia from the M1 pro-inflammatory phenotype to the M2 anti-inflammatory and reparative phenotype.

The metabolic pathways in microglia vary depending on their activation states [8]. In resting-state and M2 phenotype microglia, oxidative phosphorylation serves as the primary energy generating pathway. However, when microglia are activated to the M1 phenotype, glucose is preferentially utilized for glycolysis, even in the presence of sufficient oxygen [37]. Research has established that inhibiting the activity of metabolic enzymes involved in glycolytic reprogramming can reverse this process, promote the transition of microglia from the M1 to the M2 phenotype, mitigate the inflammatory response, and maintain the stability of the brain’s microenvironment [9,10,38,39]. Our in vitro studies revealed that the extracellular acidification rate (ECAR) significantly increased and the oxygen consumption rate (OCR) decreased in microglia following LPS treatment (Figure 5), which is consistent with previous research [40]. Moreover, NJ-1A and Nar were able to reverse the glycolytic reprogramming induced by LPS stimulation, thus facilitating the transformation of microglia from the M1 to the M2 phenotype. Our studies also demonstrated that MHY1485, an mTOR agonist, could reverse the inhibition of NJ-1A and Nar with regard to the phosphorylation level of mTOR and nullify the inhibitory effects on the secretion of NO, CCL5, and CXCL10. These findings suggest that NJ-1A and Nar can modulate the M1-to-M2 transition and suppress the expression of inflammatory and chemokine factors in activated microglia via the AKT/mTOR signaling pathway.

The role of T cells in the development of Parkinson’s disease (PD) has been attracting growing attention. Studies have demonstrated an increase in the number of CD4^+^ T cells and CD8^+^ T cells in the substantia nigra and surrounding neurons in the brains of both PD patients and mouse models [16,41], which is consistent with our in vivo findings (Figure 11). Meanwhile, the number of T cells has been shown to have a positive correlation with the number of activated microglia and the extent of neuronal loss [42]. T cells can infiltrate the brain parenchyma upon recognition of chemotactic factors (e.g., CCL2, CCL5, CXCL10) released by glial cells, such as microglia and astrocytes [30,43]. Once infiltrated, CD8^+^ T cells can differentiate into cytotoxic effector T cells upon antigen encounter. In addition to TNF-α and IFN-γ, they secrete granzyme A, B, and perforin, upregulate the expression of Fas ligand (FasL), and thus trigger neuronal death, exacerbating neuroinflammation and neurodegeneration [30,44]. Infiltrated CD4^+^ T cells, after recognizing major histocompatibility complex class II (MHC II) on the surface of microglia, can assist in the activation of CD8^+^ T cells and differentiate into T helper 1 (Th1) and T helper 17 (Th17) cells. Th1 cells secrete a large amount of IFN-γ and TNF-α, while Th17 cells mainly produce interleukin-22 (IL-22) and interleukin-17 (IL-17). These CD4^+^ T cells can further promote microglia to differentiate into the M1 pro-inflammatory type, amplifying the neuroinflammatory response and activating the Fas-FasL apoptotic signal to damage dopaminergic neurons [45,46]. In the nervous system, the high expression of MHC II is positively correlated with the severity of neuroinflammatory diseases. Clinical studies have revealed that MHC II-positive microglia accumulate in the substantia nigra of PD patients, and their numbers increase as the damage to dopaminergic neurons worsens [47]. Inhibiting MHC II expression can prevent infiltrated CD4^+^ T cells from recognizing target cell antigens, thereby blocking immune responses and alleviating damage to dopaminergic neurons [48]. In this study, we found that the expression of multiple chemokines and MHC II was significantly increased in the brains of MPTP-induced Parkinson’s disease models. NJ-1A and Nar could reduce the expression of MHC II and the release of chemokines as well as counteract the infiltration of CD4^+^ and CD8^+^ T cells into the brain parenchyma. These results suggest that the neuroprotective effects of NJ-1A and Nar may be achieved by inhibiting T cell infiltration. While NJ-1A/Nar suppressed MHC II and chemokine expression, it was unclear whether these effects were microglia-specific or also involved astrocytes or infiltrating monocytes. Lineage-specific staining or conditional knockouts could clarify cellular specificity.

Alzheimer’s disease is another common neurodegenerative disorder. Additionally, numerous reviews have shown that the roles of microglia-mediated inflammation and infiltrating T-cells in the pathological progression of Alzheimer’s disease are quite similar to those in Parkinson’s disease [49,50,51]. This suggests the possibility for the extract of *Nardostachys jatamansi* DC. and Nardosinone in the treatment of AD.

*Nardostachys jatamansi* DC., an herb that has been widely used in numerous Asian countries for centuries, has been applied in the treatment of a variety of neurological and cardiovascular disorders [20]. In China, *Nardostachys jatamansi DC.* has been used as a medicine for over 1000 years. Now, it is included in the *Chinese Pharmacopoeia* and appears in several well-known Chinese patent prescriptions, such as the Wenxin Granule and Shensong Yangxin Capsule [52]. The medicinal uses of *Nardostachys jatamansi* DC. are also well-established in India. To encourage the cultivation of *Nardostachys jatamansi* DC., which is one of the most important Indian medicinal plants, the Indian government offers a 75% subsidy for the cultivation costs for this plant [52]. The phytochemical constituents of *Nardostachys jatamansi* DC. have been studied in detail. It contains both volatile (predominantly sesquiterpenes) and non-volatile constituents, with various other compounds, such as alkaloids and essential oil-related substances [52]. This provides a guarantee for its quality control and regulation. In addition to being used as a medicine, *Nardostachys jatamansi* DC. is frequently employed as a raw material in the production of spices, food products, and cosmetics [52]. Nardosinone is used as an index for the content determination of *Nardostachys jatamansi* DC., and it is stipulated to constitute at least 0.1% of the dried, processed roots and rhizomes of this plant, according to the *Chinese Pharmacopoeia* [20]. This implies that Nardosinone may exhibit relatively low toxicity and has great potential to be developed into a new drug. In fact, as far as we know, there are currently no reports on the toxicity of *Nardostachys jatamansi* DC. and Nardosinone.

There was a study that meticulously investigated the in vivo forms of nardosinone after oral administration using the UHPLC-Q-TOF-MS technique. In total, 77 in vivo forms of Nardosinone were discovered in mice. Nardosinone was mainly excreted in urine and not detected in the feces [52]. After rats were orally given the 80% ethanol extract of *Nardostachys jatamansi* DC., nardosinone could be detected in their brains using the UHPLC-LTQ-Orbitrap-MS technique [24]. This indicated that nardosinone could cross the blood–brain barrier (BBB), especially considering that nardosinone has a relatively small molecular weight (250.33) and high solubility in methanol. Unfortunately, the study did not determine its content in the brain, so further research was needed. Our research group previously used UPLC–PDA and UHPLC–DAD/Q–TOFMS analysis techniques to analyze the degradation patterns of Nardosinone. The results showed that compared with the simulated intestinal fluid environment, nardosinone is more likely to degrade under high temperature conditions and in the simulated gastric fluid environment [20]. These research achievements are expected to provide certain valuable guidance and scientific evidence for the quality control and rational application of Nardosinone-related products.

This study used a mouse chronic model of Parkinson’s disease induced by MPTP/probenecid, which is a well-established model for studying PD [27,53]. MPTP-induced neurotoxicity in mice shares several pathological features with human PD, including dopaminergic neuron loss and neuroinflammation. However, behavioral features reminiscent of human PD are difficult to demonstrate in this model [54]. Although many MPTP-induced mouse models have shown behavioral impairments, there are also numerous studies in which the authors did not present the behavioral results, even though these MPTP-induced mouse models all exhibited pathological damage, such as the reduction of tyrosine hydroxylase (TH) [55,56,57,58]. In our study, we conducted behavioral examinations using the rotarod test on the 36th day. Compared with the control group, the MPTP group did not show any impairments; the drug-administered groups demonstrated significant therapeutic effects when compared with the model group (Figure 9H). This suggests that further behavioral assessments in alternative Parkinson’s disease models (such as α-synuclein overexpression models) are indeed required.

## 5. Conclusions

Collectively, our findings demonstrated that the NJ-1A extract derived from *Nardostachys jatamansi*, together with nardosinone, a sesquiterpenoid compound, could inhibit the M1 polarization of microglia, reverse the reprogramming of glucose metabolism, and reduce the infiltration of T cells through the AKT/mTOR signaling pathway. Consequently, these actions alleviate neurodegeneration. We provide direct evidence that reversing the glucose metabolic reprogramming in microglia and disrupting the immune hub characteristic of neurodegeneration, which exists between activated microglia and infiltrated T cells, can be effectively achieved by extracts of traditional herbal medicines, thereby conferring neuroprotective effects.

## Data Availability

The original contributions presented in this study are included in the Supplementary Materials. Further inquiries can be directed to the corresponding authors.

## Supplementary Materials

The following supporting information can be downloaded at: https://www.mdpi.com/article/10.3390/cells14090644/s1, which include: The chromatographic method of NJ-1A; Table S1: The compounds identified in NJ-1A; Table S2: Primer sequences for quantitative PCR; the original data of all bar charts; and the original pictures of Western blot, Immunofluorescence and Immunohistochemistry of this study.

## Abbreviations

The following abbreviations are used in this manuscript:

CCL	C-C motif chemokine ligand
CXCL	C-X-C motif chemokine ligan
CXCR	CXC chemokine receptor
CCR	C-C motif chemokine receptor
ECAR	extracellular acidification rate
GLUT1	glucose transporter 1
HK2	hexokinase 2
iNOS	inducible nitric oxide synthase
LPS	lipopolysaccharide
mTOR	mammalian target of rapamycin
MPTP	methyl-4-phenyl-1,2,3,6-tetrahydropyridine
NO	nitric oxide
OCR	oxygen consumption rate
PKM2	pyruvate kinase M2
ROS	reactive oxygen species
Real-time RT-PCR	Real-time reverse transcription-polymerase chain reaction
TNF-α	tumor necrosis factor-α
TH	tyrosine hydroxylase
IBA-1	ionized calcium binding adaptor molecule 1
PD	Parkinson’s disease
