# Supplementary material for: Nardostachys jatamansi Extract and Nardosinone Exert Neuroprotective Effects by Suppressing Glucose Metabolic Reprogramming and Modulating T Cell Infiltration
Source: Cells. 2025 Apr 28;14(9):644. doi: 10.3390/cells14090644 (PMC12071694; doi:10.3390/cells14090644)
Supplement: Supplementary file 1 [file cells-14-00644-s001.zip › Supplementary File S1/Table S1-the compounds identified in NJ-1A .pdf]

Supplementary Table S1: the compounds identified in NJ-1A

No.	Compounds	Formula	Retention Time (min)	Area (Max.)	Area (%)	Peak Height
1	Chlorogenic acid	C16H18O9	0.760	99177	1.18	36046
2	Chlorogenic acid	C16H18O9	2.201	223781	2.66	25148
3	Caffeic acid	C9H8O4	3.020	408842	4.86	30983
4	Linarin	C28H32O14	8.164	153724	1.83	16337
5	Desoxo-narchinol A	C12H16O2	10.293	414178	4.92	44311
6	7-oxonardosinoperoxide	C15H22O4	13.248	257721	3.06	27165
7	Nardosinonediol	C15H24O3	14.407	262523	3.12	36098
8	5H-Naphtho[1,8-bc]furan-5-one,2,2a,7,8,8a,8b-hexahydro-2,2,8a,8b-tetramethyl-, (2aR,8aS,8bR)-	C15H20O2	15.346	274721	3.26	30326
9	kanshone A	C15H22O2	15.602	352859	4.19	39184
10	Nardoaristolone B	C14H18O2	17.015	229524	2.73	24096
11	dibelon	C15H22O2	18.307	387843	4.61	44436
12	nardosinone	C15H22O3	21.128	858395	10.20	92197
13	kanshone H	C15H20O	22.838	62711	0.74	6636
14	Aristolone	$C_{15}H_{22}O$	24.737	101725	1.21	10517
