# Supplementary material for: Nardostachys jatamansi Extract and Nardosinone Exert Neuroprotective Effects by Suppressing Glucose Metabolic Reprogramming and Modulating T Cell Infiltration
Source: Cells. 2025 Apr 28;14(9):644. doi: 10.3390/cells14090644 (PMC12071694; doi:10.3390/cells14090644)
Supplement: Supplementary file 1 [file cells-14-00644-s001.zip › Supplementary File S1/Table S2-primer sequences for quantitative PCR.pdf]

Table S2. Primer Sequences for Quantitative PCR.

Gene	Primer pair（5′→3′）	Accession ID
GAPDH	F: CTTCACCACCATGGAGAAGGC<br>R: GGCATGGACTGTGGTCATGAG	XM_001476707.5
TNF-α	F: CGGGGTGATCGGTCCCCAAAG<br>R: GGAGGGCGTTGGCGCGCTGG	NM_001278601.1
IL-6	F: CCAGAGATACAAAGAAATGATGG<br>R: ACTCCAGAAGACCAGAGGAAA	NM_001314054.1
iNOS	F: GGCAGCCTGTGAGACCTTTC<br>R: CGGGGTGATCGGTCCCCAAAG	XM_006532446.3
IL-1β	F: CGCAGCAGCACATCAACAAGAGC<br>R: TGTCCTCATCCTGGAAGGTCCACG	XM_006498795.3
Ym1	F: TCACTTACACACATGAGCAAGAC<br>R: CGGTTCTGAGGAGTAGAGACCA	NM_009892.3
IL-10	F: GCTCTTACTGACTGGCATGAG<br>R: CGCAGCTCTAGGAGCATGTG	NM_010548.2
Arg1	F: GGAAGACAGCAGAGGAGGTG<br>R: TATGGTTACCCTCCCGTTGA	NM_007482.3
β-actin	F: AGAGGGAAATCGTGCGTGACATCAA<br>R: ATACCCAAGAAGGAAGGCTGGAAAA	NM_007393.5
PKM2	F: AGGATGCCGTGCTGAATG<br>R: TAGAAGAGGGGCTCCAGAGG	NM_011099.4
GLUT1	F: CAGTTCGGCTATAACACTGGTG<br>R: GCCCCCGACAGAGAAGATG	NM_011400.3
HK2	F: TCATTGTTGGCACTGGAAGC<br>R: TTGCCAGGGTTGAGAGAGAG	NM_013820.3
HIF-1α	F: GTGCTGATTTGTGAACCCATT<br>R: TCAACCCAGACATATCCACCT	NM_001313919.1
CCL2	F: GCCTGCTGTTCACAGTTGC<br>R: TCATTGGGATCATCTTGCTG	NM_011333.3
CCL5	F: CATATGGCTCGGACACCA<br>R: ACACACTTGGCGGTTCCT	NM_013653.3
CXCL10	F: CCACGTGTTG AGATCATTGCC<br>R: TCACTCCAGTTAAGGAGCCC	XM_021161764.2
CCL12	F: ATTTCCACACTTCTATGCCTCCT<br>R: ATCCAGTATGGTCCTGAAGATCA	NM_011331.3
