# Supplementary material for: Nardostachys jatamansi Extract and Nardosinone Exert Neuroprotective Effects by Suppressing Glucose Metabolic Reprogramming and Modulating T Cell Infiltration
Source: Cells. 2025 Apr 28;14(9):644. doi: 10.3390/cells14090644 (PMC12071694; doi:10.3390/cells14090644)
Supplement: Supplementary file 1 [file cells-14-00644-s001.zip › Supplementary File S1/The chromatographic method of NJ-1A.pdf]

Supplementary method: the chromatographic method of NJ-1A

The chromatographic method of NJ-1A was detailed as follows. Instrument: Waters Acquity UPLC® H class plus system (Waters Corporation, Milford, MA, USA); column: Waters ACQUITY UPLC BEH C18 (2.1 mm × 100mm, 1.7 µm); mobile phase: acetonitrile - 0.1% formic acid water; gradient elution: 0 - 1 min, 10% A; 1 - 4 min, 10% - 15% A; 4 - 6 min, 15% - 21% A; 6 - 11 min, 21% A; 11 - 13 min, 21% - 30% A; 13 - 20min, 30% - 40% A; 20 - 24 min, 40% - 50% A; 24 - 30 min, 50% - 95% A; 30 - 35 min, 95% A); flow rate: 0.3 mL/min; column temperature: 45 °C; detection wavelength: 254 nm; injection volume: 2 µL.
